# Impact of treatment with immunomodulators and tumour necrosis factor antagonists on the incidence of infectious events in patients with inflammatory bowel disease

**DOI:** 10.48101/ujms.v127.8167

**Published:** 2022-01-10

**Authors:** Per Andersson, Pontus Karling

**Affiliations:** Department of Public Health and Clinical Medicine, Umeå University, Umeå, Sweden

**Keywords:** Anti-TNF, Bowel disease, Crohn’s disease, gender differences, immunomodulators, infectious events, inflammatory ulcerative colitis

## Abstract

**Background:**

Corticosteroids, immunomodulators (IM) and tumour necrosis factor antagonists (anti-TNF) are commonly used in the treatment of inflammatory bowel disease (IBD) but they also supress the defence against infectious disease. The aim of this study was to analyse the incidence of infectious events in patients with IBD and the association to concomitant medical therapy.

**Methods:**

We performed a retrospective medical chart review of patients with IBD aged 18–65 years included in the Swedish Registry of Inflammatory Bowel Disease in the catchment area of Umeå University Hospital, Sweden. Data were collected from the period 01 January 2006, to 31 January 2019. An infectious event was defined as an outpatient prescription of antimicrobials or a positive diagnostic test for infection.

**Results:**

During a period of 5,120 observation-years, we observed 1,394 events in 593 patients. The mean number of infectious events per 100 person-years was 27.2 (standard deviation [SD]: 0.46). There were no differences in mean incidence rates between patients treated with no immunosuppression (23.0 events per 100 person-years, SD: 50.4), patients treated with IM monotherapy (27.6 events per 100 person-years, SD: 49.9), patients treated with anti-TNF monotherapy (34.3 events per 100 person-years, SD: 50.1) and patients on combination therapy (22.5 events per 100-person-years, SD: 44.2). In a multivariate logistic regression, female gender (adjusted odds ratio [AOR]: 2.24; 95% confidence interval [CI]: 1.49–3.37) and combination therapy (AOR: 3.46; 95% CI: 1.52–7.85) were associated with higher risks of infection (>32 events per 100 person years). Also, patients treated with any immunosuppression treatment for 25–75% (AOR: 2.29; 95% CI: 1.21–4.34) and for >75% (AOR: 1.93; 95% CI: 1.19–3.12) of the observation period were at higher risks compared to patients treated with immunosuppression <25% of the observation period.

**Conclusion:**

We observed no significant difference in risk for infections between patients on monotherapy with IM or anti-TNF and patients with low use of immunosuppression, but there was a significant risk for combination therapy.

## Introduction

Inflammatory bowel disease (IBD) is a group of chronic conditions of presumed autoimmune aetiology, that includes ulcerative colitis (UC) and Crohn’s disease (CD) [1, 2]. IBD is characterised by intermittent inflammation of the gastrointestinal tract with varying extension, severity and frequency of relapse.

The aim of the treatment of IBD is to induce and sustain remission. The treatment strategy varies with the subtype, extension, localisation and severity of the disease, frequency of relapse and response of earlier treatments [3, 4]. Traditional therapies consist mainly of 5-aminosalicylic acid, corticosteroids and antimetabolite immunomodulators (IM) such as thiopurines and methotrexate. In more recent years, biological agents, mainly in the form of tumour necrosis factor antagonists (anti-TNF), integrin antagonists (vedolizumab) and interleukin antagonists (ustekinumab), have emerged as alternatives and complements for patients with moderate-severe disease, or when treatment goals have not been reached by traditional means [3, 4].

The treatment of IBD with drugs that interact with the immune system increases the risk for infectious disease [5, 6]. For example, systemic corticosteroids, especially in higher doses, significantly increase the risks of serious [7] and opportunistic [8] infections. Furthermore, thiopurines are associated with an increased risk of opportunistic infections in patients with IBD [8], as well as with overall infection in patients with UC [5]. But, the risk of infection when on treatment with anti-TNFs is controversial. While an observational, post-marketing study indicates an increased risk of serious infections compared to non-biological treatment [7], recent meta-analyses have not found an increased risk of serious infections with anti-TNF treatment compared to placebo [6, 9, 10]. However, the risk of overall [6] and opportunistic [6, 11] infections seems to increase with anti-TNF treatment.

The combination of anti-TNF and IM is often used in clinical practice. The combination of infliximab and azathioprine was found to result in higher rates of steroid-free remission than that for infliximab monotherapy [12, 13]. However, the knowledge of the safety of combination therapy is not fully understood. One retrospective cohort study has indicated no increased risk for serious infections compared to anti-TNF monotherapy [14], while another study demonstrated an increased risk [15]. However, the risk of opportunistic infection seems to be higher with combination therapy than anti-TNF monotherapy or thiopurine monotherapy [14, 15].

Therefore, we believe that more knowledge of the potential risks of combination therapy is needed. Previous studies have mainly focused on serious and opportunistic infections. However, the rates of any infection can also affect the adherence to treatment [16], which might impair the long-term outcome of IBD [17]. The aim of this study was to report the frequency of infectious events in patients with IBD that have led to an intervention from the healthcare system, and to study to what extent treatment with steroids, IM and anti-TNF is associated with these events. The present study focused on both factors associated with each infectious event and on the frequency of infectious events in each patient.

## Methods

### Study population

The study population was derived from the Swedish Registry of Inflammatory Bowel Disease, SWIBREG – a nationwide registry covering 59% of the Swedish IBD population [18]. Patients aged 18–65 years on 01 February 2019, and who were treated at the Department of Medicine at Umeå University Hospital (NUS) within the period of 2006–2019, were included. Inclusion was made on 01 January 2006, for prevalent cases which were 18 years of age or older. Younger patients were included from when they turned 18 years of age, and incident cases from the date of the first positive finding on endoscopy, either macro- or microscopically. Patients were excluded if they lacked a UC or CD diagnosis, had been treated at NUS only as children, resided outside of Västerbotten County or had blocked their medical charts. Patients were also censored from the date of diagnosis of malignancy, organ transplantation surgery, or from the date patients with UC underwent colectomy. The total number of patients (>18 years old) with IBD treated at the clinic was approximately 900 [19].

### Definitions

An infectious event was defined as an outpatient prescription of antibiotics or antimicrobials due to infectious disease and/or a positive diagnostic test for infectious disease (bacteria, virus or fungi). Antibiotics prescribed due to IBD related complications, such as fistulas or abscesses, were not considered as events, and neither were antibiotics prescribed prophylactically, post-operatively or due to primary sclerosing cholangitis. Multiple events on the same date were registered as a single event as were the prescriptions and positive tests from different dates but were clearly linked. Diagnostic tests were not considered as events if the result was described as borderline or commensal flora. To exclude positive cultures due to normal colonisation flora, skin and urine cultures were considered positive only if the patient received infection treatment. When calculating incidence rates (events per 100 person-years) only patients observed more than 1 year on a treatment were included. Due to small numbers of patients and events on several drugs, incidence rate was only calculated for no immunosuppressive treatment, steroid treatment, IM treatment (thiopurines or methotrexate), anti-TNF treatment and combination treatment (IM + anti-TNF).

With the term ‘immunosuppressive treatment’, we included treatment with systemic prednisolone, thiopurines, methotrexate, anti-TNFs, anti-integrins and anti-interleukins. With the term IM, we included thiopurines (azathioprine, 6-mercaptopurine and 6-thioguanine) and methotrexate. Combination therapy was defined as concomitant treatment with an IM and an anti-TNF. Budesonide was not considered as a systemic steroid in this study.

The exposure of infliximab, vedolizumab or ustekinumab was considered to last for 8 weeks since the last infusion. The exposure of thiopurines and adalimumab was considered to last for 4 weeks since the last administration. Other treatments were considered to last until the last day of administration.

To determine patients at risk for an infectious disease, we set the primary outcome at a cut-off at the upper quartile of the incidence rate of infectious events. Secondary outcomes were defined as prescriptions of antibiotics, antivirals and antimycotics, and positive tests for bacterial, viral and mycotic infection.

### Data collection

Data collection was performed by both authors retrospectively by reviewing medical charts of the Department of Internal Medicine at NUS dated 01 January 2006 to 31 January 2019. These charts have access to prescriptions and microbiological diagnostics from all outpatient care provided by the Region of Västerbotten County. Data concerning date of diagnosis, number of years treated at NUS, Montreal classification [20], surgery due to IBD, medical treatment for IBD, and diagnostics and outpatient antimicrobial treatment for infectious disease were collected.

The characteristics of the events were registered along with concomitant medication for IBD or immunosuppressant medication due to other reasons. Data concerning whether the event was related to surgery was also collected.

### Statistics

Descriptive statistics were used to characterise the study population and the infectious events. Categorical variables were analysed using Chi-squared test, and presented as frequencies and percentages. Incidence rate was both calculated as means and medians. Students *t*-test was used to compare incidence rate for specific treatments. Parameters that were not normally distributed were also analysed using Mann-Whitney test, and were presented as median and first and third quartiles. A *P*-value < 0.05 was considered statistically significant, and we did not correct for multiple testing. For patients observed 3 years or more, a multivariate logistic regression was used. The dependent variable was the risk for infectious events (upper quartile of the incidence rate of infectious disease) and the independent variables were age, gender, CD, and treatment of immunosuppressive drugs. The impact of immunosuppressive drugs was tested in one model for duration on immunosuppressive treatment (<25%, 25–75% and >75% of the observation period) and in a second model for the use of IM, anti-TNF and combination therapy (>75% of the observation period) with low use of immunosuppressive treatment (<25% of the observation period) as reference.

Analyses were performed using Statistical Package for Social Sciences (SPSS) Statistics 25.0 (IBM Corporation, Armonk, NY).

### Ethical considerations

The study did not include any interventional procedures. The study was approved by the ethical committee in Umeå, Dnr 2016-27431, and by the Stockholm ethics committee, 2016/191-31.

## Results

### Study population

**Figure 1:**
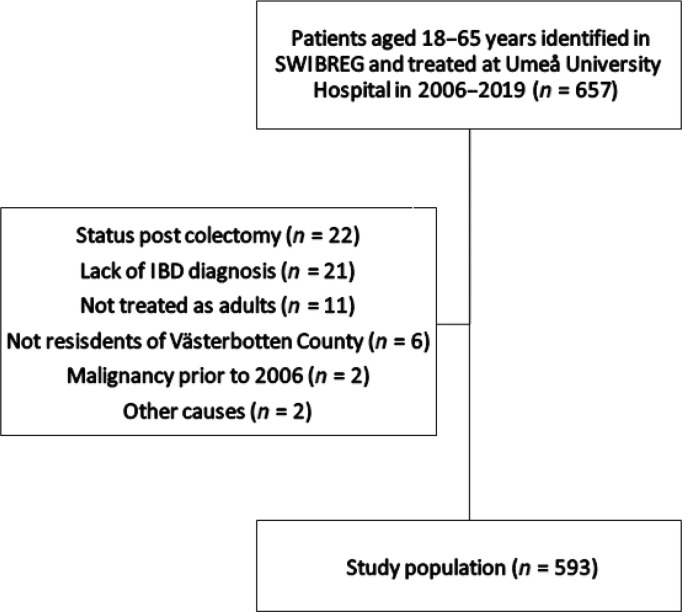
The recruitment of the study population.

The study population is described in Table 1, and the number of included and excluded patients are shown in Figure 1.

**Table 1:** Characteristics of patients included in the study.

	All (*n* = 593)	UC (*n* = 379)	CD (*n* = 195)
Follow up duration, years, median (Q₁–Q₃)	9 (5–13)	9 (5–13)	10 (4–13)
Age 01 February 2019, years, median (Q₁–Q₃)	42 (34–53)	43 (35–53)	40 (32–52)
Age at diagnosis, years, median (Q₁–Q₃)	26 (19–35)	26 (20–35)	24.5 (18–33)
Male sex, *n* (%)	329 (55.5)	213 (56.2)	107 (54.9)
UC, *n* (%)	379 (63.9)	-	-
CD, *n* (%)	195 (32.9)	-	-
Unclassified colitis, *n* (%)	19 (3.2)	-	-
**Montreal classification:**			
A1, *n* (%)	93 (15.7)	54 (14.6)	39 (20.7)
A2, *n* (%)	397 (66.9)	267 (72.0)	122 (64.9)
A3, *n* (%)	84 (14.2)	50 (13.2)	27 (14.4)
E1, *n* (%)	-	49 (12.9)	-
E2, *n* (%)	-	116 (30.6)	-
E3, *n* (%)	-	206 (54.4)	-
L1, *n* (%)	-	-	44 (22.6)
L2, *n* (%)	-	-	72 (36.9)
L3, *n* (%)	-	-	73 (37.4)
L4, *n* (%)	-	-	10 (5.1)
B1, *n* (%)	-	-	102 (52.3)
B2, *n* (%)	-	-	46 (23.6)
B3, *n* (%)	-	-	44 (22.6)
p, *n* (%)	-	-	43 (22.1)
**At any time treated with:**			
Five-ASA, *n* (%)	485 (81.8)	359 (94.7)	107 (54.9)
Budesonide, *n* (%)	144 (24.3)	39 (10.3)	103 (52.8)
Systemic prednisolone, *n* (%)	314 (53.0)	205 (54.1)	103 (52.8)
Thiopurines, *n* (%)	283 (47.7)	143 (37.7)	137 (70.3)
Methotrexate, *n* (%)	34 (5.7)	15 (4.0)	19 (9.7)
Infliximab, *n* (%)	109 (18.4)	51 (13.5)	56 (28.7)
Adalimumab, *n* (%)	67 (11.3)	17 (4.5)	50 (25.6)
Other anti-TNF, *n* (%)	8 (1.3)	6 (1.6)	2 (1.0)
Vedoluzimab, *n* (%)	13 (2.2)	9 (2.4)	4 (2.1)
Ustekinumab, *n* (%)	11 (1.9)	0 (0.0)	11 (5.6)
Combination therapy (IM + anti-TNF), *n* (%)	120 (20.2)	48 (12.7)	71 (36.4)

*n*: number; *UC*: ulcerative colitis; CD: Crohn’s disease; y: years; Q_1_: first quartile; Q_3_: third quartile; A1: age at diagnosis ≤16 years; A2: age at diagnosis 17–40 years; A3: age at diagnosis >40 years; E1: ulcerative proctitis; E2: left-sided colitis; E3: extensive colitis; L1: terminal ileum; L2: colon; L3: ileocolonic; L4: upper gastrointestinal tract; B1: non-stricturing nonpenetrating; B2: structuring; B3: penetrating; p: perianal; Five-ASA: 5-aminosalicylic acid; IM: immunomodulator; anti-TNF: tumour necrosis factor antagonist.

*Note*: The sum of the numbers of treatments exceeds the number of patients because each patient might have received several different treatments and treatment combinations. Nineteen patients were classified as unclassified IBD.

Patients with CD had been treated significantly more frequent with IM (*P* < 0.001), anti-TNF (*P* < 0.001) and combination therapy (*P* < 0.001) than those with UC (Table 1). IM monotherapy (patients who had received IM but never anti-TNF) was also more common among CD than UC patients (32.3% vs. 24.0%, *P* = 0.034). There was no difference in the proportion of patients who had at least once been exposed to systemic corticosteroids between patients with UC and CD (*P* = 0.773)

Compared to patients not receiving immunosuppressive treatment, patients receiving IM monotherapy more often had CD (40.6% vs. 19.9%, *P* < 0.001), and had more severe disease characteristics in terms of extensive UC (60.0% vs. 40.2%, *P* = 0.004) and stricturing and/or penetrating CD (40.3% vs. 8.6%, *P* = 0.001). Those who received combination therapy more often had CD (59.2% vs. 19.9%, *P* < 0.001) and had more severe disease characteristics in terms of age of onset ≤16 years (24.8% vs. 13.8%, *P* = 0.032), extensive UC (79.2% vs. 40.2%, *P* < 0.001) and stricturing and/or penetrating CD (73.2% vs. 8.6%, *P* < 0.001) than those not receiving immunosuppressive treatment.

Furthermore, compared to patients receiving IM monotherapy, those receiving combination therapy more often had CD (*P* = 0.002), and had more severe disease characteristics in terms of age of onset ≤16 years (*P* = 0.006), extensive UC (*P* = 0.020) and stricturing and/or penetrating CD (*P* < 0.001). There was no difference in the proportion of patients that at least once had received systemic corticosteroids between the groups receiving IM monotherapy and combination therapy (71.0% vs. 72.5%, *P* = 0.780).

### Infectious events overall

During a total follow-up time of 5,120 years, we identified 1,465 events in 593 patients. Of these, 71 events occurred post-surgery, leaving 1,394 events included in the analysis. The infectious events are characterised in Table 2. The type of microorganisms on culture/polymerase chain reaction (PCR) and the type of antimicrobial drug prescribed at the infectious events are shown in Tables 3 and 4. Overall, the mean number of infectious events per 100 person-years was 27.2 (standard deviation [SD]: 0.46) and the median number of infectious events per 100 person-years was 11 (25th–75th percentile: 0–32 per 100 person-years). Women had significant more events than men (38.1 versus 18.3 mean events per 100 perso-years, *P* < 0.001). Escherichia coli (E. coli) infection (*P* < 0.001) and the prescription of antibiotics typically used for urinary tract infection (pivmecallinam and nitrofurantoin, *P* < 0.001) were significantly higher in women than in men. Also, candida albicans infection (*P* < 0.001) and treatment with fluconazole (*P* < 0.001) were significantly higher in women. Flucloxacillin (*P* < 0.001) and ciprofloxacin (*P* < 0.001) were significantly more often prescribed for men. Nearly half of all infectious events were not associated with any immunosuppressive treatment. The most common treatment that was associated with an infectious event was thiopurines. There were no significant differences in mean incidence rate of infectious events between the different treatments except for steroid treatment that had a significantly higher mean incidence rate than no immunosuppressive treatment (*P* < 0.001), treatment with IM (*P* = 0.001) and treatment with combination therapy (*P* = 0.003) (Table 2). However, there was no significant difference in the median number of events between steroid treatment and other treatments. E. coli (*P* = 0.011) and staphylococcus aureus (*P* < 0.002) were significantly more often diagnosed in patient with no immunosuppression whereas cytomegalovirus (*P* < 0.001) was diagnosed significantly more often in patients on immunosuppressive treatment.

**Table 2:** Characterisation of medical treatment for IBD at the time of event and event characteristics.

Ongoing treatment at the time of event	Number of events, *n* (%) *n* = 1,394	Number of patients with events *n* = 358	Mean events per 100 Person-years (SD)
No immunosuppression	663 (47.4)	223	23.0 (50.4)
Systemic corticosteroids	116 (8.3)	44	69.5 (127)^a^
Thiopurines	469 (33.5)	136	27.6 (49.9)^b^
Methotrexate	31 (2.2)	11	NA
Anti-TNF	247 (17.7)	70	34.3 (50.1)^c^
Vedolizumab	11 (0.8)	6	NA
Ustekinumab	7 (0.5)	3	NA
Combination therapy (Immunomodulators + anti-TNF)	140 (10.0)	48	22.5 (44.2)
Combination therapy + systemic corticosteroids	17 (1.2)	7	NA
**Characteristic of events**	**Number of events, *n* (%) *n* = 1,394**	**Number of patients with events *n* = 358**	
Prescription of:			
Antibiotics	1,039 (70.9)	319	-
Antivirals	100 (6.8)	47	-
Antimycotics	158 (10.8)	55	-
Positive test for:			
Bacterial infection	311 (21.2)	134	-
Viral infection	77 (5.3)	48	-
Fungal infection	46 (3.1)	26	-

*TNF*: tumour necrosis factor; SD: standard deviation.

a Includes combination therapy with other immunosuppressives.

b Only monotherapy with thiopurines or methotrexate.

c Only monotherapy with anti-TNF.

**Table 3:** Type of microorganisms diagnosed at infectious events.

Bacteria	*N*
Bacteroides fragilis	1 (1)
Borrelia burgdorferi	1 (0)
Campylobacter jejuni	3 (2)
Chlamydia pneumoniae	3 (3)
Chlamydia trachomatis	7 (3)
Citrobacter freundii	3 (3)
Clostridioides difficile	3 (0)
Enterobacteriaceae	4 (2)
Enterococcus faecalis	8 (5)
Enterohemorrhagic Escherichia coli	2 (2)
Escherichia coli	62 (23)
Francisella tularensis	1 (0)
Haemophilus influenza	15 (4)
Helicobacter pylori	4 (3)
Klebsiella oxytoca	2 (1)
Klebsiella pneumoniae	7 (5)
Moraxella catarrhalis	4 (1)
Mycobacterium tuberculosis	1 (1)
Mycoplasma genitalum	1 (0)
Mycoplasma pneumoniae	3 (0)
Neisseria gonorrhoeae	1 (1)
Neisseria meningtidis	1 (0)
Prevotella bivia	2 (1)
Proteus mirabilis	1 (0)
Proteus vulgaris	1 (0)
Pseudomonas aurogimosa	4 (3)
Salmonella enterica	1 (1)
Serrata marcesens	3 (0)
Staphylococcus aureus	55 (18)
Staphylococcus, coagulase-negative	6 (3)
Staphylococcus epidermis	2 (2)
Staphylococcus hominis	4 (2)
Staphylococcus intermedius	1 (1)
Staphylococcus lugdunesis	3 (1)
Staphylococcus saprophyticus	5 (0)
Streptococcus group A	12 (6)
Streptococcus group B	2 (2)
Streptococcus group C	1 (0)
Streptococcus group G	6 (3)
Streptococcus dysgalactiace	1 (1)
Streptococcus anginosus	1 (0)
Streptococcus pneumoniae	9 (5)
Yersinia enterocolitica	1 (1)
**Virus**	***N***
Cytomegalovirus	21 (20)
Ebstein-Barr virus	4 (1)
Echovirus	1 (0)
Enterovirus	3 (1)
Herpes Simplex virus 1	5 (1)
Herpes Simplex virus 2	2 (1)
Influenza A	5 (3)
Influenza AH3	1 (0)
Metapneumovirus	2 (1)
Norvovirus	2 (0)
Parainfluenza virus	2 (2)
Puumula virus	7 (6)
Respiratory syncytial virus	1 (1)
Rhinovirus	2 (2)
Rotavirus	1 (0)
Saprovirus	2 (1)
Variciella	2 (1)
**Fungi**	***N***
Acremonium	1 (1)
Aspergillus fumigatus	1 (0)
Candida albicans	25 (13)
Candida glabrate	1 (1)
Candida paropsilosis	2 (1)
Candida tropicalis	1 (0)
Tricophyton rubrum	6 (3)

*N* = number of events (number of events on immunosuppression treatment).

**Table 4:** Type of antimicrobial drug used at infectious events.

Antimicrobial drug	*N*
Aciclovir	55 (36)
Amoxicillin	58 (23)
Amoxicillin/Clavulanic acid	27 (17)
Azitromycin	7 (2)
Cephadroxil	33 (15)
Cephibuthen	6 (1)
Ciprofloxacin	71 (41)
Clarithromycin	5 (4)
Clindamycin	47 (24)
Doxycycline	150 (67)
Erythromycin	3 (2)
Ethambutol	2 (0)
Fluconazole	118 (61)
Flucloxacillin	155 (82)
Isoniazide	3 (3)
Itraconazole	3 (0)
Levofloxacin	4 (2)
Metronidazole	17 (12)
Nitrofurantoin	59 (18)
Norfloxacin	3 (2)
Nystatin	30 (19)
Oseltamivir	3 (2)
Penicillin V	272 (152)
Pivmecillinam	96 (46)
Rifampicin	3 (3)
Terbinafine	7 (4)
Trimethoprim	33 (17)
Trimethoprim/Sulfametoxazole	7 (3)
Valacyclovir	40 (24)
Valganciclovir	2 (2)
Variconazole	1 (1)

*N* = number of events (number of events on immunosuppression treatment).

Seventy-seven percent (*n* = 1,080) of the events were related to bacterial infection (prescription of antibiotics and/or positive bacterial test), while 11.8% (*n* = 16) and 12.2% (*n* = 17) of the events were related to viral and fungal infections respectively. The most common infections diagnosed with culture/PCR were E. coli, staphylococcus aureus, candida albicans, cytomegalovirus, haemophilus influenza, streptococcus pneumonia, klebsiella pneumonia, and chlamydia trachomatis (Table 3). Overall, only one patient was diagnosed with mycobacterium tuberculosis and that patient was diagnosed during combination therapy with azathioprine and infliximab.

### Infectious events per patient

Sixty percent of the patients had at least one event during the follow-up time. Among patients who at some time had been treated with immunosuppressive medication, 36.7% of the events occurred during a period when they did not receive these treatments.

The median number of infectious events per year was significantly higher among patients with CD compared to patients with UC (15 per 100 person-years; 25th–75th percentile 0–42 vs. 9 per 100 person-years; 25th–75th percentile 0–0.30; *P* = 0.039). The difference was, however, not statistically significant when comparing CD and UC within each treatment group (IM and anti-TNF monotherapy or combination therapy).

Patients treated with immunosuppressive treatment for more than 75% of the observation period (median 17 vs. 9 events per 100 person-years; *P* = 0.011) and patient treated with immunosuppressive treatment between 25–75% of the observation time had more events (20 vs. 9 events per 100 person-years; *P* = 0.027) and had significantly higher incidence rates compared to patients with immunosuppressive treatment less than 25% of the observation period. Patients treated with IM monotherapy (median 15 vs. 9 events per 100 person-years; *P* = 0.42), with anti-TNF monotherapy (median 11 vs. 9 events per 100 person-years; *P* = 0.60) and with combination therapy (median 25 vs. 9 events per 100 person-years; *P* = 0.094) for more than 75% of the observation period did not significantly differ in incidence rate to patients with immunosuppression for less than 25% of the observation period.

In a multivariate logistic regression model assessing different length of immunosuppressive treatment, patients treated with immunosuppression for 25–75% of the observation period and patients treated more than 75% of the observation period had a significant but similar higher risk for infections events compared to patient treated less than 25% of the observation period (Table 5). Patients on combination therapy for more than 75% of the observation period were also at higher risks but patients on monotherapy were not (Table 6).

**Table 5:** Patient with inflammatory bowel disease observed for 3 years and more (*n* = 527).

	Univariate odds ratio (95% CI)	Multivariate odds ratio (95% CI)
Age	0.99 (0.98–1.01)	0.99 (0.98–1.011)
Female gender (*n* = 232)	2.06 (1.39–3.06)	2.24 (1.49–3.37)
Crohn’s disease (*n* = 170)	1.79 (1.20–2.69)	1.39 (0.89–2.18)
**Duration on immunosuppressive treatment:**		
>75% or the observation period (*n* = 182)	2.00 (1.31–3.06)	1.93 (1.19–3.12)
25–75% of the observation period (*n* = 56)	2.44 (1.32–4.51)	2.29 (1.21–4.34)
Reference: <25% of the observation period (*n* = 289)		

CI: confidence interval.

Logistic regression with dependent variable high risk for infectious disease (>32 event per 100 person-years) and independent variables age, gender, Crohn’s disease and duration of immunosuppressive treatment during the observation period.

**Table 6:** Patient with inflammatory bowel disease observed for 3 years or more (*n* = 427).

	Univariate odds ratio (95% CI)	Multivariate odds ratio (95% CI)
Age	0.99 (0.97–1.01)	1.00 (0.98–1.02)
Female gender (*n* = 183)	2.07 (1.31–3.27)	2.30 (1.42–3.73)
Crohn’s disease (*n* = 122)	1.19 (1.17–3.04)	1.54 (0.90–2.65)
**Immunosuppressive treatment**		
Immunomodulators (*n* = 85)	1.17 (0.65–2.11)	1.19 (0.63–2.24)
Anti-TNF (*n* = 16)	1.36 (0.42–4.36)	1.17 (0.34–4.00)
Combination therapy (*n* = 37)	3.46 (1.70–7.03)	3.46 (1.52–7.85)
Reference: Immunosuppression <25% of the observation period (*n* = 289)		

*CI*: confidence interval; anti-TNF: tumour necrosis factor antagonists.

Logistic regression with dependent variable high risk for infectious disease (>32 event per 100 person-years) and independent variables age, gender, Crohn’s disease and treatment with immunomodulators, anti-TNF and combination therapy (>75% of the observation period).

## Discussion

In this retrospective observational study in patients with IBD under 65 years of age, we found an overall low incidence of infectious events during the study period. Approximately 60% of the patients during a median follow up of 9 years had at least one infectious event defined by either having been prescribed antimicrobial treatment or a positive test for bacteria, virus or fungi. Nearly half of all infectious events in our study occurred when the patient was not on any immunosuppressive therapy. The concomitant treatment associated with most infectious event was thiopurines but only 1 of 10 infectious events was associated with combination therapy.

As suspected, the present study showed that the risk of an infection was significantly higher among IBD patients treated with immunosuppression compared to patients with a low use of immunosuppression. However, we could not detect any difference in incidence rate of infectious events between patients treated with IM and anti-TNF monotherapy compared to patients with a low use of immunosuppression. Only combination therapy with IM and anti-TNF showed an increased risk in the multivariate analysis. Interestingly, patients treated for 25–75% of the observation time with immunosuppression were at the same risk as patients treated for more than75% of the observation time.

Previous studies have shown an increased risk for overall infectious diseases during treatment with IM or anti-TNF [5, 6]. Earlier observations have also stated that patients treated with combination therapy are at higher risks than patients treated with monotherapy [14, 15]. Differences in study population and study design may partly explain the different outcomes in our study compared to earlier studies regarding monotherapy of IM and anti-TNF. For example, in our study we had a narrow definition of an infectious event, and we did our best to exclude events related to complication of IBD (post-operative infection, perianal abscesses and primary sclerosing cholangitis).

As stated by Kirchgesner et al. [15], most observational studies analysing the association of IBD treatment and infection rates do not properly consider the use of corticosteroids, which is a major risk factor for infections [5, 8]. In the present study, only 8.3% of the events occurred during systemic corticosteroid treatment. The mean incidence rate but not the median incidence rate was higher in patients on systemic steroids compared to other treatments. However, our study was underpowered to assess the risk of steroid therapy for infectious event considering factors such as combination with other therapies and dose effects.

The increased risk for infection for patients on combination therapy shown in our study support that IM and anti-TNF therapy should be carefully controlled in patients with IBD. For example, one should aim to optimise 6-thoguanine nucleotide levels so they do not exceed therapeutic intervals. Also, in a patient with combination therapy who reached remission, a lower dose of IM could be considered [21]. Further, to prevent antibodies against infliximab a 6-thigoguanine level of 125 pmol/8 × 10^8^/L RBC may be sufficient [22].

In a large Swedish, nationwide register-based cohort study, the incidence rate of a first severe infection (equal to hospital admission) was significantly higher compared to the general population (2.7 vs. 1.1 per 100 person-years) [23]. In our study, we assessed the overall number of infectious events in our patients and we did not specifically define the severity of the infection. Neither did we include parameters of inflammation such as C-reactive protein and albumin.

Of special concern when planning immunosuppressive treatment, is the risk of primary or reactivated mycobacterium tuberculosis infection [24]. However, in the Western world, tuberculosis is still uncommon. For example, only 0.2% of the patients treated with anti-TNF for rheumatoid arthritis were diagnosed with tuberculosis after treatment [25], and in a prospective study from Japan that followed 570 patients for 1 year, no case of active tuberculosis was observed [26]. In line with this, in the present study only one patient was diagnosed with tuberculosis during the study and that patient was diagnosed on combination therapy.

The mean incidence rate in female patients with IBD was two-fold that of the mean incidence rate for men in our study. Especially, there were events related to lower urinary tract infection and vaginal candida infections that were prominent among women but seldom among men. Also, in the general population, there is a higher rate of prescription of antibiotics in primary care for women in the age 16–54 years compared to men [27], which indicates that the gender differences in infection rate seen in our study might not be related to IBD.

The strength of our study lies in that the medical charts give access to all prescriptions and microbiologic diagnostics from all departments in Västerbotten County; this should ensure an inclusion of the vast majority of events.

The major drawback of the study is the inability of an observational study to exclude the impact of confounders. One major difference between the treatment groups is the severity of the IBD itself. We have taken measures to exclude events directly related to the patients’ IBD, for example, those connected to fistulas, primary sclerosing cholangitis and surgery. However, there is a risk that not all of these events were excluded, due to lack of documentation of the causes of prescriptions and diagnostic testing in the medical records. This could increase the event rate among patients with a more aggressive disease, and consequently, receiving more aggressive medical treatment.

The treatment strategy could also impact the vigilance of signs of infection among both professionals and patients, thus leading to increased use of diagnostic tests and antimicrobials in patients with assumed immunocompromising treatments.

Our study only focused on infectious events that lead to an intervention from the medical care system, and we did not measure self-limited infectious events such as common cold and other common virus infections. Furthermore, we lack information of vaccinations in our patients, and one can assume that the proportion of patients who had had vaccinations for influenza and pneumococcus is probably higher in the patient treated with IM and biologics. Furthermore, a substantial proportion of the patients at our clinic were at the time of the study not registered in SWIBREG and therefore, there could be a selection bias in the study.

Finally, the data on the infectious events for each patient in our study could be influenced by variations in the time of exposure to, compliance to and doses of the different treatments.

In conclusion, the overall frequency of infectious events in patients with IBD was low in our study. Patients who received immunosuppression therapy and patient on combination therapy (IM+ anti TNF) have significantly higher risks for an infectious event than patients with a low use of immunosuppressive treatment. We cannot exclude that a heightened disease activity in these patients may partly explain the increased risk for infection. However, there was no significant difference in risk between patients on monotherapy with IM or anti-TNF, and patient with low use of immunosuppression.

## References

[1] Baumgart DC, Sandborn WJ. Crohn’s disease. Lancet. 2012;380:1590–605. doi: 10.1016/S0140-6736(12)60026-9 22914295

[2] Ford AC, Moayyedi P, Hanauer SB. Ulcerative colitis. BMJ. 2013;346:f432. doi: 10.1136/bmj.f432 23386404

[3] Harbord M, Eliakim R, Bettenworth D, Karmiris K, Katsanos K, Kopylov U, et al. Third European evidence-based consensus on diagnosis and management of ulcerative colitis. Part 2: current management. J Crohns Colitis. 2017;11:769–84. doi: 10.1093/ecco-jcc/jjx009 28513805

[4] Gomollon F, Dignass A, Annese V, Tilg H, Van Assche G, Lindsay JO, et al. 3rd European evidence-based consensus on the diagnosis and management of Crohn’s disease 2016: Part 1: diagnosis and medical management. J Crohns Colitis. 2017;11:3–25. doi: 10.1093/ecco-jcc/jjw168 27660341

[5] Lichtenstein GR, Rutgeerts P, Sandborn WJ, Sands BE, Diamond RH, Blank M, et al. A pooled analysis of infections, malignancy, and mortality in infliximab- and immunomodulator-treated adult patients with inflammatory bowel disease. Am J Gastroenterol. 2012;107:1051–63. doi: 10.1038/ajg.2012.89 22613901 PMC3390465

[6] Bonovas S, Fiorino G, Allocca M, Lytras T, Nikolopoulos GK, Peyrin-Biroulet L, et al. Biologic therapies and risk of infection and malignancy in patients with inflammatory bowel disease: a systematic review and network meta-analysis. Clin Gastroenterol Hepatol. 2016;14:1385–97.e10. doi: 10.1016/j.cgh.2016.04.039 27189910

[7] Lichtenstein GR, Feagan BG, Cohen RD, Salzberg BA, Diamond RH, Price S, et al. Serious infection and mortality in patients with Crohn’s disease: more than 5 years of follow-up in the TREAT registry. Am J Gastroenterol. 2012;107:1409–22. doi: 10.1038/ajg.2012.218 22890223 PMC3438468

[8] Toruner M, Loftus EV, Jr, Harmsen WS, Zinsmeister AR, Orenstein R, Sandborn WJ, et al. Risk factors for opportunistic infections in patients with inflammatory bowel disease. Gastroenterology. 2008;134:929–36. doi: 10.1053/j.gastro.2008.01.012 18294633

[9] Shah ED, Farida JP, Siegel CA, Chong K, Melmed GY. Risk for overall infection with anti-TNF and anti-integrin agents used in IBD: a systematic review and meta-analysis. Inflamm Bowel Dis. 2017;23:570–7. doi: 10.1097/MIB.0000000000001049 28230558

[10] Wheat CL, Ko CW, Clark-Snustad K, Grembowski D, Thornton TA, Devine B. Inflammatory Bowel Disease (IBD) pharmacotherapy and the risk of serious infection: a systematic review and network meta-analysis. BMC Gastroenterol. 2017;17:52. doi: 10.1186/s12876-017-0602-0 28407755 PMC5391579

[11] Ford AC, Peyrin-Biroulet L. Opportunistic infections with anti-tumor necrosis factor-alpha therapy in inflammatory bowel disease: meta-analysis of randomized controlled trials. Am J Gastroenterol. 2013;108:1268–76. doi: 10.1038/ajg.2013.138 23649185

[12] Colombel JF, Reinisch W, Mantzaris GJ, Kornbluth A, Rutgeerts P, Tang KL, et al. Randomised clinical trial: deep remission in biologic and immunomodulator naive patients with Crohn’s disease – a SONIC post hoc analysis. Aliment Pharmacol Ther. 2015;41:734–46. doi: 10.1111/apt.13139 25728587

[13] Panaccione R, Ghosh S, Middleton S, Marquez JR, Scott BB, Flint L, et al. Combination therapy with infliximab and azathioprine is superior to monotherapy with either agent in ulcerative colitis. Gastroenterology. 2014;146:392–400.e3. doi: 10.1053/j.gastro.2013.10.052 24512909

[14] Osterman MT, Haynes K, Delzell E, Zhang J, Bewtra M, Brensinger CM, et al. Effectiveness and safety of immunomodulators with anti-tumor necrosis factor therapy in Crohn’s disease. Clin Gastroenterol Hepatol. 2015;13:1293–301.e5; quiz e70, e72. doi: 10.1016/j.cgh.2015.02.017 25724699 PMC4475667

[15] Kirchgesner J, Lemaitre M, Carrat F, Zureik M, Carbonnel F, Dray-Spira R. Risk of serious and opportunistic infections associated with treatment of inflammatory bowel diseases. Gastroenterology. 2018;155:337–46.e10. doi: 10.1053/j.gastro.2018.04.012 29655835

[16] Martelli L, Lopez A, Strobel S, Danese S, Roblin X, Baumann C, et al. Adherence to infliximab therapy in inflammatory bowel disease patients in a real-life setting. J Dig Dis. 2017;18:566–73. doi: 10.1111/1751-2980.12539 28858439

[17] Perry J, Chen A, Kariyawasam V, Collins G, Choong C, Teh WL, et al. Medication non-adherence in inflammatory bowel diseases is associated with disability. Intest Res. 2018;16:571–8. doi: 10.5217/ir.2018.00033 30301333 PMC6223449

[18] Ludvigsson JF, Andersson M, Bengtsson J, Eberhardson M, Fagerberg UL, Grip O, et al. Swedish Inflammatory Bowel Disease Register (SWIBREG) – a nationwide quality register. Scand J Gastroenterol. 2019;54:1089–101. doi: 10.1080/00365521.2019.1660799 31498717

[19] Lindhagen S, Karling P. A more frequent disease monitorering but no increased disease activity in patients with inflammatory bowel disease during the first year of the SARS-CoV-2 pandemic. A retrospective study. Scand J Gastroenterol. 2021;56:1–6. doi: 10.1080/00365521.2021.1993328 34699290

[20] Satsangi J, Silverberg MS, Vermeire S, Colombel JF. The Montreal classification of inflammatory bowel disease: controversies, consensus, and implications. Gut. 2006;55:749–53. doi: 10.1136/gut.2005.082909 16698746 PMC1856208

[21] Roblin X, Boschetti G, Williet N, Nancey S, Marotte H, Berger A, et al. Azathioprine dose reduction in inflammatory bowel disease patients on combination therapy: an open-label, prospective and randomised clinical trial. Aliment Pharmacol Ther. 2017;46:142–9. doi: 10.1111/apt.14106 28449228

[22] Yarur AJ, Kubiliun MJ, Czul F, Sussman DA, Quintero MA, Jain A, et al. Concentrations of 6-thioguanine nucleotide correlate with trough levels of infliximab in patients with inflammatory bowel disease on combination therapy. Clin Gastroenterol Hepatol. 2015;13:1118–24.e3. doi: 10.1016/j.cgh.2014.12.026 25562796

[23] Ludvigsson JF, Holmgren J, Grip O, Halfvarson J, Askling J, Sachs MC, et al. Adult-onset inflammatory bowel disease and rate of serious infections compared to the general population: a nationwide register-based cohort study 2002–2017. Scand J Gastroenterol. 2021;56:1152–62. doi: 10.1080/00365521.2021.1924259 34369254

[24] Debeuckelaere C, De Munter P, Van Bleyenbergh P, De Wever W, Van Assche G, Rutgeerts P, et al. Tuberculosis infection following anti-TNF therapy in inflammatory bowel disease, despite negative screening. J Crohns Colitis. 2014;8:550–7. doi: 10.1016/j.crohns.2013.11.008 24295645

[25] Cantini F, Niccoli L, Goletti D. Tuberculosis risk in patients treated with non-anti-tumor necrosis factor-alpha (TNF-alpha) targeted biologics and recently licensed TNF-alpha inhibitors: data from clinical trials and national registries. J Rheumatol Suppl. 2014;91:56–64. doi: 10.3899/jrheum.140103 24789001

[26] Naganuma M, Kunisaki R, Yoshimura N, Takeuchi Y, Watanabe M. A prospective analysis of the incidence of and risk factors for opportunistic infections in patients with inflammatory bowel disease. J Gastroenterol. 2013;48:595–600. doi: 10.1007/s00535-012-0686-9 23053426

[27] Schroder W, Sommer H, Gladstone BP, Foschi F, Hellman J, Evengard B, et al. Gender differences in antibiotic prescribing in the community: a systematic review and meta-analysis. J Antimicrob Chemother. 2016;71:1800–6. doi: 10.1093/jac/dkw054 27040304

